# His Bundle Pacing Improves Left Ventricular Function in Patients with Bradyarrhythmia or Tachy-Brady Syndrome and Permanent Atrial Fibrillation: A Retrospective Analysis

**DOI:** 10.3390/jcm14092860

**Published:** 2025-04-22

**Authors:** Patrycja Paluszkiewicz, Adrian Martuszewski, Jacek Smereka, Jacek Gajek

**Affiliations:** 1 Department of Emergency Medical Service, Wroclaw Medical University, ul. Parkowa 34, 51-616 Wrocław, Poland; 2 Division of Environmental Health and Occupational Medicine, Department of Population Health, Wroclaw Medical University, Mikulicza-Radeckiego 7, 50-368 Wrocław, Poland; 3 Medical Faculty, Wrocław University of Science and Technology, 50-368 Wrocław, Poland

**Keywords:** His bundle pacing, heart failure, permanent atrial fibrillation, bradycardia, tachy-brady syndrome, sinus node disease

## Abstract

**Background**: Permanent atrial fibrillation (AF) frequently coexists with heart failure (HF), leading to structural remodeling and progressive sinus node dysfunction. As the condition advances, bradyarrhythmia or tachy-brady syndrome may develop. Right ventricular pacing and cardiac resynchronization therapy may impair left ventricular function due to non-physiological ventricular activation. His bundle pacing (HBP) offers a more physiological alternative. This study evaluates HBP’s impact on left ventricular function in patients with bradyarrhythmia or tachy-brady syndrome and permanent AF. **Methods**: A retrospective analysis included 41 patients with HF who underwent HBP implantation due to bradyarrhythmia or tachy-brady syndrome in permanent AF. LVEF, LVEDD, and MR were assessed before and after implantation, alongside the impact of comorbidities (e.g., ischemic heart disease and chronic kidney disease) and pharmacotherapy (digoxin, metoprolol, and mineralocorticoid receptor antagonists). Statistical analyses included the Wilcoxon test (LVEF and MR), paired Student’s *t*-test (LVEDD), Spearman’s correlation, and linear regression. Significance was set at *p* < 0.05. **Results**: HBP significantly improved LVEF (median increase: 14.58%; *p* < 0.001) and reduced LVEDD (mean reduction: 5.41 ± 1.30 mm; *p* < 0.001). MR severity also decreased (*p* < 0.001). Patients with lower baseline LVEF showed greater improvement in this parameter after HBP (ρ = −0.671, *p* < 0.001). Only chronic kidney disease was associated with a lower likelihood of MR improvement (*p* = 0.0486). **Conclusions**: HBP improves left ventricular function and reduces MR severity in patients with permanent AF and bradyarrhythmia or tachy-brady syndrome. A low baseline LVEF was the strongest predictor of subsequent improvement. Further studies are needed to confirm long-term benefits.

## 1. Introduction

Permanent atrial fibrillation (AF) is frequently associated with heart failure (HF), forming a vicious cycle of mutual structural and functional interactions. Management remains challenging due to atrial remodeling, a high risk of AF recurrence, and the presence of comorbidities that significantly worsen the prognosis [1,2,3]. Progressive changes in the sinus node resulting from atrial remodeling may lead to bradyarrhythmias and tachy-brady syndrome [4]. In this patient population, rhythm control often fails, and the limited effectiveness of pharmacological therapy creates the need for alternative strategies.

Permanent AF increases the risk of developing atrial functional mitral regurgitation (AFMR), a condition with a pathophysiology distinct from classical secondary functional mitral regurgitation. Chronic left atrial volume overload leads to dilation of the mitral annulus and impaired leaflet coaptation, without significant structural abnormalities of the valve itself. Despite its high prevalence (up to 60%), AFMR often remains undiagnosed. Although the restoration of sinus rhythm has been shown to reduce the degree of mitral regurgitation, there is a lack of data regarding the impact of His bundle pacing (HBP) on this parameter [5]. AFMR is characterized by preserved left ventricular geometry and function, moderate to severe left atrial enlargement, and mitral annular dilation without significant leaflet restriction. The lack of a uniform definition hinders research on its prevalence, prognosis, and management. Standardizing the definition is essential for better understanding and optimal clinical care [6]. A key therapeutic goal in this patient population is to achieve ventricular rate control while preserving physiological systolic synchrony.

In cases of inadequate pharmacological rate control, treatment may involve atrioventricular node ablation (AVNA) followed by pacemaker implantation. This strategy ensures complete ventricular rate control and when implemented with conduction system pacing may help preserve physiological ventricular activation [7,8]. Traditional right ventricular pacing (RVP) produces non-physiological activation and electrical dyssynchrony, which may worsen left ventricular function and increase the risk of HF progression. An alternative approach is cardiac resynchronization therapy (CRT). However, its effectiveness depends on venous anatomy and the positioning of the left ventricular lead, and a substantial proportion of patients fail to respond to this therapy [9].

HBP represents an alternative to conventional approaches, facilitating ventricular activation via the heart’s native conduction system and ensuring a more physiological excitation pattern. HBP is recommended for patients in whom CRT has failed, those with atrioventricular block and a LVEF greater than 40% who require more than 20% ventricular pacing, individuals at high risk of pacing-induced cardiomyopathy (PICM), those requiring a “pace-and-ablate” strategy, and some patients with intraventricular conduction disturbances in whom pacing near the His bundle may overcome a pre-existing left bundle branch block (LBBB). The 2021 ESC guidelines highlight the need for further research into the clinical benefits of HBP compared with RVP and CRT [10]. Unlike RVP, this technique preserves contraction synchrony, enhances LVEF, and may even facilitate reverse cardiac remodeling without exacerbating HF progression. HBP may be particularly beneficial for patients in whom CRT was ineffective or could not be performed [11].

This study aimed to evaluate the impact of His bundle lead implantation on left ventricular function in patients with HF and bradyarrhythmia or tachy-brady syndrome during permanent AF.

## 2. Materials and Methods

The analysis was conducted on a study group comprising 41 patients who underwent HBP due to bradyarrhythmia or tachy-brady syndrome in the course of permanent AF. All patients had permanent AF, defined as continuous AF with no attempts to restore sinus rhythm and no documented reversions during follow-up. The indication for HBP was symptomatic bradyarrhythmia or episodes of rapid AF alternating with long post-conversion pauses within the AF rhythm, consistent with tachy–brady syndrome in the context of permanent AF. All patients had a narrow QRS complex on baseline ECG (<120 ms). None of the patients underwent AVNA. Ventricular pacing was performed using single-chamber devices programmed in VVI or VVIR mode, as no patients were candidates for dual-chamber pacing. All patients with HF with reduced LVEF received guideline-directed medical therapy, including beta-blockers or MRAs. Information on the use and dosage of MRAs was available and included in the dataset. However, data regarding other classes of diuretics (e.g., loop or thiazide diuretics) were not collected in a standardized manner and were therefore not analyzed.

The control group comprised the same patients prior to pacing. HBP in the study group was performed by an experienced cardiologist and echocardiographer, a recognized authority in the field of cardiac electrophysiology, with extensive clinical and research experience (J.G.). The study group underwent an initial assessment 1–3 months after HBP, and an ultrasound follow-up was performed after 6 months. The local Bioethics Committee approved this study, and all patients provided written informed consent before undergoing the procedure. Clinical data included demographic characteristics, comorbidities, laboratory parameters, and pharmacological treatments. Table 1 presents key baseline data for the included patients.

Echocardiographic parameters, including LVEDD, LVEF, and the degree of MR, were used to assess the outcomes of HBP. LVEF was measured using the biplane method of disks (modified Simpson’s rule), based on the manual tracing of endocardial borders in the apical four- and two-chamber views, following current echocardiographic guidelines. All examinations were performed by the same experienced echocardiographer using a consistent acquisition protocol and ultrasound system. Although formal blinding was not feasible due to the retrospective nature of the study, this approach ensured consistency and minimized interobserver variability. The LVEF measurement was rounded to the nearest whole number. Table 2 presents the PICO framework, outlining the study population, intervention, comparator, and outcome measures.

The primary outcome measure was the percentage change in LVEF following implantation. Descriptive statistics included calculating means, medians, standard deviations, and quartiles for quantitative variables depending on the normality of data distribution, while categorical variables were presented as percentages. The Shapiro–Wilk test was used to assess the normality of continuous variables. Only left ventricular end-diastolic diameter (LVEDD) before and after pacing met the assumptions of normal distribution (*p* > 0.05), which justified the use of the paired Student’s *t*-test. All other continuous variables, including LVEF and laboratory parameters (such as glucose and creatinine levels), did not follow a normal distribution. Qualitative variables, including comorbidities and pharmacotherapy (including presence of HF, diabetes, or use of digoxin, beta-blockers, and mineralocorticoid receptor antagonists), were not tested for normality. To analyze differences between values before and after implantation, the Wilcoxon test was used for LVEF and the degree of MR, while the paired Student’s *t*-test was applied for LVEDD. The statistical analysis also included Spearman’s correlation in the context of LVEF and Pearson’s correlation in the context of normally distributed LVEDD. Linear regression analysis was conducted to identify predictors of LVEF improvement. Additionally, LVEF improvement was compared in subgroups of patients with and without HT, CKD, IHD, MI, and DM and based on the baseline degree of MR. Group comparisons were performed using Student’s *t*-test and the Kruskal–Wallis test for ordinal variables.

Statistical analysis was conducted using RStudio 2024.12.0 Build 467 with the dplyr, ggplot2, stats, and pwr packages. Statistical power was analyzed using the pwr.t.test function, calculating the minimum required sample size and the post hoc power of the conducted tests. Minimum sample size tests were performed for the study. All analyses were performed at a significance level of 0.05.

## 3. Results

### 3.1. Changes in LVEF, LVEDD, and MR After His Bundle Pacing

Statistical analysis of patients with HF, bradyarrhythmia, and tachy-brady syndrome in persistent AF revealed that HBP significantly increased LVEF from 55% to 60% (IQR: 60–65%), reduced LVEDD from 55.6 ± 1.81 mm to 50.2 ± 1.60 mm, and decreased MR severity compared to pre-pacing values. Although the median baseline LVEF was 55%, all patients fulfilled clinical criteria for HF. The study cohort included patients with heart failure with preserved (HFpEF), mildly reduced (HFmrEF), and reduced ejection fraction (HFrEF), in accordance with ESC definitions. The observed increase in LVEF demonstrated a functional recovery and reverse remodeling in the HF population. While specific pacing percentages were not systematically collected, all patients had clinical indications requiring permanent pacing support due to bradyarrhythmia or tachy-brady syndrome in the setting of permanent AF. Therefore, the observed improvements in LVEF and LVEDD can be attributed to the sustained effect of HBP pacing in this population. The results are presented in Table 3.

**Figure 1:**
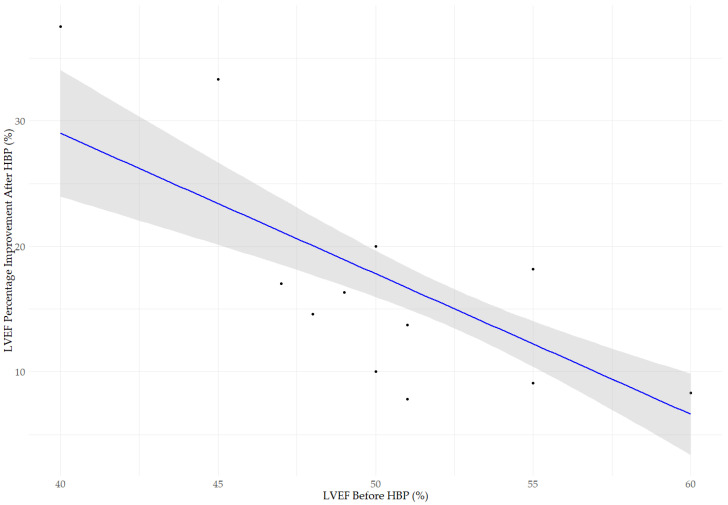
Relationship between baseline left ventricular ejection fraction (LVEF before HBP) and the percentage improvement in LVEF, calculated as (LVEF after HBP − LVEF before HBP)/LVEF before HBP) × 100%. The blue line represents the linear regression model, while the gray area indicates the 95% confidence interval. A statistically significant negative correlation was observed between baseline LVEF (LVEF before HBP) and the degree of improvement after HBP (LVEF percentage improvement after HBP; Spearman’s rho, ρ = −0.671, *p* < 0.001), indicating that patients with lower baseline LVEF experienced more pronounced improvement in systolic function following implantation. The median LVEF increased from 55% (IQR: 50–55%) to 60% (IQR: 60–65%) (Wilcoxon rank-sum test, *p* < 0.001). The improvement in LVEF varied significantly (Kruskal–Wallis test, χ^2^ = 20.965, df = 2, *p* < 0.001).

The scatter plot shown in Figure 1 illustrates the relationship between the pre-implantation LVEF and the percentage improvement in LVEF. The LVEF percentage improvement was defined as the relative change compared to the baseline value, calculated as ((LVEF after HBP − LVEF before HBP)/LVEF before HBP) × 100%. This allows for proportional comparison across patients with different degrees of baseline systolic function. A statistically significant negative correlation was observed (Spearman’s rho, ρ = −0.671, *p* < 0.001), suggesting that patients with lower pre-procedural LVEF experienced greater improvement after implantation. The median LVEF before implantation was 55% (IQR: 50–55%), increasing to 60% (IQR: 60–65%) after the procedure (Wilcoxon rank-sum test, *p* < 0.001). Subgroup analysis identified statistically significant differences in LVEF improvement between the LVEF groups (Kruskal–Wallis test, χ^2^ = 20.965, *df* = 2, *p* < 0.001).

**Figure 2:**
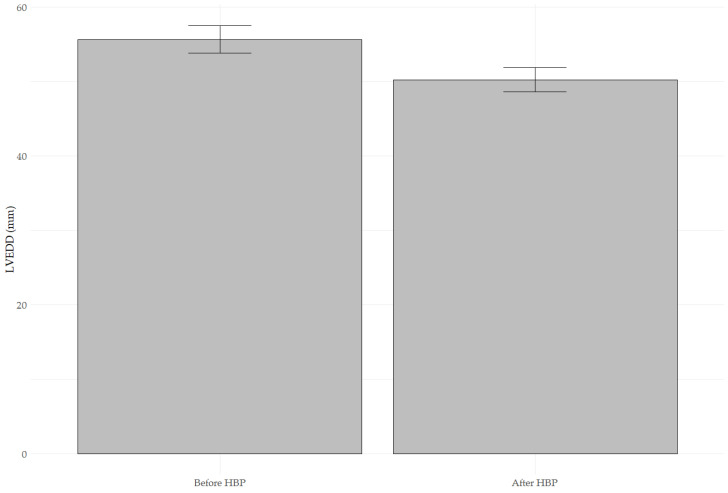
The bar plot compares the mean LVEDD values measured before implantation (LVEDD Before HBP) and after implantation (LVEDD After HBP). A significant reduction in mean LVEDD was observed after implantation (55.63 ± 1.81 mm vs. 50.22 ± 1.60 mm; paired *t*-test, t = 26.601, *p* < 0.001). The error bars present standard deviations. The effect size (Cohen’s d = 3.13) indicates a clinically significant change.

Figure 2 presents a bar plot comparing mean LVEDD values before and after HBP. A statistically significant reduction in mean LVEDD was observed after implantation (55.63 ± 1.81 mm vs. 50.22 ± 1.60 mm, paired *t*-test, *p* < 0.001). The mean difference was −5.41 ± 1.30 mm, indicating a substantial decrease in left ventricular size. The effect size (Cohen’s d = 3.13) suggests this change’s very high clinical significance.

**Figure 3:**
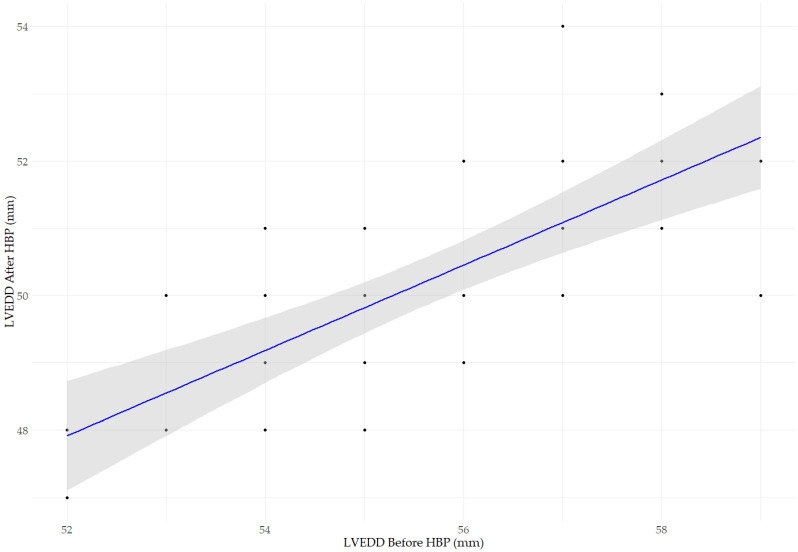
The scatter plot illustrates the relationship between LVEDD before and after HBP implantation. A significant positive correlation was observed (Pearson’s, r = 0.716, *p* < 0.001; R^2^ = 0.512, *p* < 0.001), indicating that post-implantation LVEDD values are partially predictable based on baseline measurements. The regression coefficient (β = 0.633, *p* < 0.001) suggests that a larger baseline LVEDD is associated with a larger LVEDD after HBP implantation, although the reduction is not strictly linear. The blue line represents the linear regression model, while the gray area indicates the 95% confidence interval.

Figure 3 presents a scatter plot illustrating the relationship between LVEDD before and after HBP. We observed a significant positive correlation (R^2^ = 0.512, *p* < 0.001), indicating that post-implantation LVEDD values are partially predictable based on baseline measurements. The blue line represents the linear regression model, while the gray area indicates the 95% confidence interval. The regression coefficient (*β* = 0.633, *p* < 0.001) suggests that a more significant baseline LVEDD is associated with a higher post-implantation LVEDD, although the reduction is not strictly linear.

Changes in MR before and after HBP were also analyzed. The MR scale included the following points: 0—without, 1—mild, 2—moderate, and 3—severe. The Wilcoxon test demonstrated a statistically significant reduction in MR post-implantation (*p* < 0.001). The MR transition analysis (Table 4) revealed that 26 out of 41 patients (63.4%) experienced a reduction in MR severity. One patient experienced a complete resolution of mitral regurgitation.

**Figure 4:**
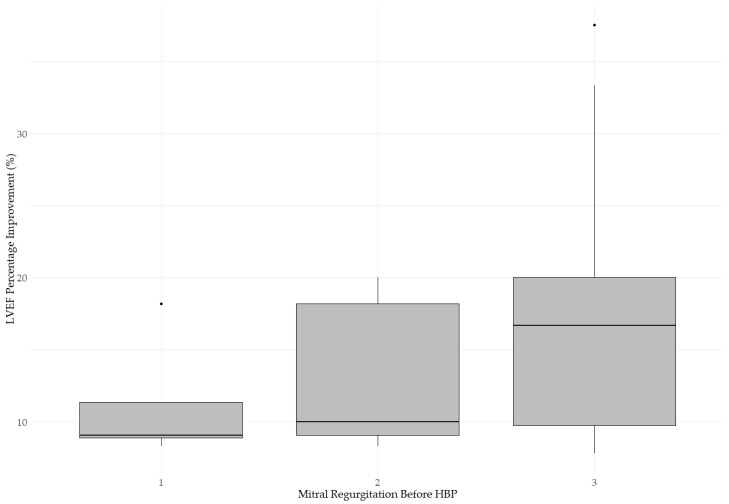
Change in LVEF based on baseline MR severity. The median LVEF improvement was 9.09% (IQR: 8.90–11.36%) in patients with mild MR before HBP, 10.00% (IQR: 9.09–18.18%) in patients with moderate MR before HBP, and 16.7% (IQR: 9.77–20.00%) in patients with severe MR before HBP. The Kruskal–Wallis test revealed no significant difference (*p* = 0.1738), indicating no impact of baseline MR on LVEF improvement.

The change in LVEF was also assessed based on the baseline severity of MR (Figure 4). The box plot illustrates LVEF improvement (%) after HBP in three patient groups categorized by pre-procedural MR severity. The median LVEF improvement was 9.09% (IQR: 8.90–11.36%) in patients with mild MR before HBP, 10.00% (IQR: 9.09–18.18%) in patients with moderate MR before HBP and 16.7% (IQR: 9.77–20.00%) in patients with severe MR before HBP. The Kruskal–Wallis test revealed no significant differences in LVEF improvement between the groups (*p* = 0.1738), suggesting that baseline MR severity did not influence the magnitude of systolic function improvement and that HBP may provide benefits regardless of initial MR grade.

### 3.2. Impact of Medications and Comorbidities on Changes in Echocardiographic Parameters After Pacing

HBP did not show a significant impact of medications (digoxin, metoprolol, or MRA) or comorbidities (IHD, DM, HT, or CKD) on LVEF improvement or LVEDD reduction. Similarly, the analysis of medications and comorbidities on MR changes revealed that none of these factors significantly influenced the final MR severity, except for patients with CKD, who had a lower likelihood of MR improvement (*p* = 0.0486). No significant correlation was found between metoprolol/MRA doses and MR improvement. The detailed results are presented in Table 5.

### 3.3. Minimum Sample Size

Sample size calculations indicated that the minimum number of patients required to detect a significant difference in LVEF was 4, confirming that the sample size of 41 patients was fully adequate. The post hoc power analysis demonstrated 100% power, further supporting the result’s reliability.

### 3.4. Summary of Results

HBP effectively improves left ventricular function in patients with bradyarrhythmias and tachy-brady syndrome associated with persistent AF. The degree of improvement in LVEF was more pronounced in patients with lower baseline LVEF values, indicating the potential for greater benefit in those with more advanced systolic dysfunction.

## 4. Discussion

Permanent cardiac pacing remains a cornerstone therapy for irreversible conduction disturbances and symptomatic bradycardia. Although RVP is widely used, it is associated with risks of dyssynchrony, PICM, HF progression, increased mortality, and hospitalization rates, particularly in patients with reduced LVEF. These adverse effects are thought to result from asymmetric myocardial hypertrophy, worsening mitral regurgitation, and impaired systolic function. Studies have shown that HBP leads to greater improvements in LVEF, LVEDD, and NYHA class compared with RVP, especially in patients with atrioventricular (AV) block and permanent AF following AVNA. The most pronounced benefits were observed when the conduction block was located at the AV node, whereas infranodal blocks could often be overcome using non-selective HBP. HBP appears particularly effective in patients with LVEF between 35% and 52%, as well as in those with a high burden of ventricular pacing [12,13,14]. Additionally, AV conduction-optimized HBP provides superior cardiac function compared to RVP with a ventricular pacing omitting algorithm in patients with prolonged PR interval [15].

Patients with HFrEF who are dependent on ventricular pacing require CRT, but the presence of AF is associated with worse prognosis. HBP represents an alternative approach, but the limited data demonstrating the superiority of physiological over non-physiological pacing emphasize the need for further research. The study by Ma et al. [16] compared HBP with BVP in 52 patients with permanent AF, bradyarrhythmia, and HFrEF. Both groups showed improvements in LVEF and reductions in LVEDD, but the effect was significantly greater in the HBP group (LVEF: +12.25% vs. +8.07%, *p* = 0.013; LVEDD: −10.13 mm vs. −6.00 mm, *p* = 0.003). MR also decreased in both groups, although the difference between them was not statistically significant (*p* = 0.521). In the CRT group, a significant QRS prolongation was observed, which may exacerbate dyssynchrony and impair valvular function. HBP, by preserving the physiological sequence of activation, enhances systolic synchrony and promotes reversible left ventricular remodeling, which may lead to improved mitral and tricuspid valve function. HBP appears particularly effective in patients who do not respond to CRT, which accounts for approximately 20 to 30 percent of cases, or in those for whom coronary sinus lead implantation is technically challenging [17]. Similar to our study, the greatest improvement was observed in patients with lower baseline LVEF (≤30%), suggesting a higher therapeutic potential of this pacing modality in this specific subgroup [18].

AFMR is most commonly observed in patients with AF and HFpEF, where the coexistence of these conditions is associated with pronounced atrial remodeling, elevated levels of natriuretic peptides, and worse clinical outcomes. Although MR improvement in this population has traditionally been linked to the restoration of sinus rhythm, our findings suggest that MR reduction may also occur in the setting of permanent AF, due to favorable left ventricular remodeling induced by HBP. Reestablishing physiological ventricular activation through HBP, along with LVEDD reduction and improvement in mitral apparatus geometry, promotes more effective leaflet coaptation, regardless of atrial rhythm. This is the first study to suggest that ventricular activation regularization via HBP may significantly reduce the severity of MR in this patient population. This effect may support global cardiac remodeling and improvement in overall cardiac function. Due to the lack of dedicated guidelines for the management of AFMR, detailed echocardiographic assessment, especially three-dimensional analysis of the mitral annulus, remains essential [19,20]. Epidemiological data indicate that even mild AFMR is associated with worse prognosis in HFpEF, which positions HBP as a potential therapeutic and preventive strategy in this group of patients [6]. Currently, no specific guidelines exist for the treatment of AFMR, and management is largely based on approaches used for classical secondary MR. Treatment should be individualized, taking into account the possibility of restoring and maintaining sinus rhythm. In patients with chronic AF, physiological pacing should be considered, whereas in more advanced cases, surgical intervention, such as annuloplasty, may be necessary. Given the growing number of patients with AF and HFpEF, further research is essential to better understand the pathophysiology of AFMR and to optimize both diagnostic and therapeutic strategies [21]. Upadhyay et al. [22] evaluated the effects of non-selective HBP on MR and systolic dysfunction in patients with LVEF below 50%. A significant reduction in MR was observed in most patients, accompanied by left ventricular volume reduction, improved contractility, QRS narrowing, and favorable changes in mitral valve geometry. In patients who did not experience MR improvement, no significant changes were seen in left ventricular geometry or systolic function. Improvement in MR following HBP was associated with better ventricular remodeling, a decrease in mitral annular area, and reduced leaflet tethering forces. Our findings are consistent with these observations, suggesting that HBP contributes to MR improvement through structural remodeling and physiological ventricular activation. Further studies are needed to evaluate the long-term impact of this pacing strategy on MR.

HBP is used in patients with symptomatic bradycardia and AF to maintain an appropriate heart rate and ensure physiological myocardial depolarization. Qu et al. [23] demonstrated the superiority of HBP over RVP and CRT in the treatment of bradycardia and conduction disorders without reversible causes. As early as 2006, a case was reported of a patient with permanent AF, bradycardia, and HFrEF, in whom HBP led to improvement in cardiac function. The first reports of temporary HBP in dogs date back to 1967, and pacing maintained for 27 months confirmed the durability and clinical efficacy of this technique [24,25]. Soral et al. [26] compared the effects of HBP in patients with LVEF below 50% and those with normal LVEF, without performing AVNA. In patients with reduced LVEF, significant improvements were observed in LVEF, LVEDD, and NYHA class, along with favorable cardiac remodeling. In contrast, patients with normal LVEF showed no significant changes and no deterioration in cardiac function. This study, like ours, confirms the efficacy of HBP even without AVNA, provided there is a high burden of ventricular pacing (greater than 40%). Compared with RVP, HBP has been shown to reduce all-cause mortality and heart failure hospitalizations (HFHs), even in patients with advanced AV block and normal or mildly reduced LVEF [27]. In patients with HFrEF, persistent AF, a high burden of RVP, and a narrow QRS complex, a dual-chamber ICD with permanent HBP is superior to a single-chamber ICD. It is the only strategy that allows preservation of the native narrow QRS, thereby preventing adverse left ventricular remodeling and complications associated with conventional pacing. HBP reduces bradycardia episodes, allows for β-blocker up-titration, and contributes to improved clinical outcomes [28].

Beta-blockers are currently considered a cornerstone of pharmacological therapy in patients with HFrEF, including those undergoing conduction system pacing, in accordance with current ESC and HRS guidelines. However, an increasing body of evidence suggests that the benefits of beta-blockers in patients with permanent AF and HF may be limited [29]. A meta-analysis by Kotecha et al. [30] demonstrated that in patients with AF, beta-blockers used for rate control did not reduce mortality and were associated with a higher rate of HFHs. Similar conclusions were drawn in a meta-analysis by Rienstra et al. [31], which showed no survival benefit from beta-blockers in patients with HF and coexisting AF, in contrast to a clear protective effect in those with sinus rhythm. Proposed mechanisms include an increased risk of bradycardia and pause-related arrhythmias in AF patients treated with beta-blockers, which may worsen cardiac function. It has also been suggested that the beneficial effects of beta-blockers may be primarily mediated via the sinus node, limiting their efficacy in AF, where ventricular rate depends on atrioventricular conduction. Moreover, optimal rate control strategies in AF may differ from those applied in sinus rhythm. Available data suggest that a moderate resting heart rate (70–89 bpm) may be associated with better outcomes than more restrictive approaches. In this context, conduction system pacing, including HBP, may offer therapeutic value by providing rhythm stability and preventing bradycardic pauses, which could indirectly improve beta-blocker tolerability. Potential strategies to improve outcomes also include pacemaker implantation and/or pulmonary vein isolation in selected patients to restore sinus rhythm [32,33].

AF and sinus node dysfunction (SND) frequently coexist in elderly patients with structural heart disease and atrial myopathy. Nearly half of patients receiving a pacemaker due to SND also have a diagnosis of AF, and clinically this overlap often manifests as tachy-brady syndrome, characterized by alternating episodes of rapid AF and sinus bradycardia or prolonged pauses. This syndrome complicates rhythm control strategies and is associated with worse prognosis. Chronic AF may further contribute to sinus node remodeling and dysfunction, while the presence of electroanatomical abnormalities in the course of SND may predispose to AF, creating a vicious cycle. In selected patients with drug-refractory AF and rapid ventricular response, AVNA may be considered to achieve rate control; however, this strategy necessitates permanent pacemaker implantation and does not restore sinus rhythm. The restoration of sinus rhythm has been shown to improve sinus node function, suggesting a bidirectional relationship. The successful ablation of paroxysmal AF in patients with pauses exceeding three seconds improves sinus node function. In patients with symptomatic SND, choosing an appropriate pacing mode is crucial to reducing the risk of AF recurrence. Physiological pacing that minimizes ventricular stimulation is recommended [34]. It has been shown that most patients with tachy-brady syndrome maintain sinus rhythm despite a high level of pacemaker use. Nearly half of patients exhibit a high burden of ventricular pacing (≥50%) [35]. In this context, a high percentage of ventricular pacing further increases the clinical relevance of HBP, which—by preserving physiological activation—can significantly support and improve left ventricular function.

Despite its many advantages, HBP also has limitations. It is a technically demanding pacing method that requires operator experience and often higher pacing thresholds. In cases with a low percentage of ventricular pacing (<20%), the benefits of HBP in improving cardiac function are minimal, aside from reducing the risk of tricuspid regurgitation. HBP may not be effective in patients with distal conduction block. Further investigations are required to elucidate the benefits and limitations of this technique [24].

## 5. Limitations

Our single center retrospective study has several important limitations. The lack of a control group treated with conventional RVP/CRT makes it difficult to directly compare the effectiveness of HBP with standard pacing techniques. Additionally, data on pharmacotherapy and comorbidities may influence the observed changes in left ventricular function, requiring further analysis in larger populations. Comprehensive data on diuretic therapy other than MRAs were not available. Diuretic use may influence the patient’s fluid balance and preload conditions, which can, in turn, affect echocardiographic parameters such as LVEDD or the severity of MR. This factor could not be adequately controlled for in the present analysis. Due to the retrospective design of the study, data on natriuretic peptides (BNPs or NT-proBNPs) were not uniformly available and were therefore not included in the analysis. As these biomarkers are clinically relevant indicators of HF severity and response to therapy, their inclusion would have provided additional insight and should be considered in future prospective studies. Finally, the follow-up period may be insufficient to fully assess the long-term impact of HBP on left ventricular remodeling and patient prognosis. Further studies in larger patient cohorts may better define the long-term effects of this therapeutic strategy.

## 6. Conclusions

HBP in patients with bradyarrhythmia and tachy-brady syndrome in the setting of permanent AF leads to a significant improvement in LVEF, a reduction in LVEDD, and a decrease in the severity of MR. The strongest predictor of LVEF improvement was its baseline value, with our findings indicating that patients with more severely reduced systolic function prior to implantation experienced the greatest improvement following HBP. This suggests that individuals with lower baseline LVEF may benefit most from this pacing strategy. A larger LVEDD before implantation was also correlated with better improvement in left ventricular function. The presence of IHD had no significant impact on left ventricular function recovery.

The analyses confirm that HBP may serve as an effective strategy for enhancing systolic function in a carefully selected patient population. HBP is a technically challenging intervention, which often requires a high pacing threshold that increases over time. Further research involving larger cohorts and longer follow-up periods is necessary to better establish the long-term benefits and feasibility of this therapeutic approach.

## Figures and Tables

**Table 1:** Baseline data of the sample (n = 41).

Parameters	
Female ^a^	27/68.85
Age [years] ^c^	71 (68–75)
Medications ^a^	
Digoxin usage	18/43.90
Dose of metoprolol:	
12.5 mg	1/2.44
25.0 mg	5/12.20
50.0 mg	8/19.51
100.0 mg	17/41.46
150.0 mg	2/4.88
200.0 mg	8/19.51
Dose of MRA:	
without	25/60.98
12.5 mg	1/2.44
25.0 mg	12/29.27
50.0 mg	3/7.31
Laboratory parameters	
Hb [g/dL] ^b^	14.19 ± 1.05
K+ [mmol/L] ^b^	4.46 ± 0.38
Glucose [mmol/L] ^c^	115.00 (102.00–137.00)
Creatinine [mg/dL] ^c^	0.90 (0.80–1.10)
Comorbidities ^a^	
HT	36/87.80
DM	13/31.71
CKD	3/7.32
IHD	10/24.39
MI	5/12.20
Echocardiographic parameters	
Baseline LVEDD [mm] ^b^	55.63 ± 1.81
Baseline LVEF [%] ^c^	55.00 (50.00–55.00)
Baseline MR: ^a^	
without	0/0
mild	4/9.76
moderate	21/51.22
severe	16/39.02

^a^—presented as number/percentage; ^b^—presented as mean ± standard deviation; ^c^—presented as median (Q1–Q3). Abbreviations: CKD, chronic kidney disease; DM, diabetes mellitus; LVEF, left ventricular ejection fraction; Hb, hemoglobin; HT, hypertension; IHD, ischemic heart disease; K+, potassium; LVEDD, left ventricular end-diastolic diameter; MI, myocardial infarction; MR, mitral regurgitation; MRA, mineralocorticoid receptor antagonist.

**Table 2:** PICO framework.

Population (P)	41 patients with heart failure, bradyarrhythmia, or tachy-brady syndrome in the course of persistent AF with indications for pacemaker implantation.
Intervention (I)	Application of HBP.
Comparison (C)	Same patients before HBP.
Outcome (O)	LVEF, LVEDD, and degree of MR.

Abbreviations: AF, atrial fibrillation; LVEF, left ventricular ejection fraction; HBP, His bundle pacing; LVEDD, left ventricular end-diastolic diameter; MR, mitral regurgitation.

**Table 3:** Echocardiographic parameters before and after pacing (n = 41).

Echocardiographic Parameters	Before HBP	After HBP	*p*-Value
LVEDD [mm] ^b^	55.63 ± 1.81	50.22 ± 1.60	<0.001
LVEF [%] ^c^	55.00 (50.00–55.00)	60.00 (60.00–65.00)	<0.001
LVEF increase [%] ^c^	–	14.58 (9.09–20.00)	–
MR: ^a^			<0.001
without	0/0	1/2.44	
mild	4/9.76	28/68.29	
moderate	21/51.22	10/24.39	
severe	16/39.02	2/4.88	

^a^—presented as number/percentage; ^b^—presented as mean ± standard deviation; ^c^—presented as median (Q1–Q3). Abbreviations: LVEF, left ventricular ejection fraction; HBP, His bundle pacing; LVEDD, left ventricular end-diastolic diameter; MR, mitral regurgitation.

**Table 4:** Changes in MR severity before and after HBP. The MR scale included the following points: 0—without, 1—mild, 2—moderate, and 3—severe.

	MR 0 After ^a^	MR 1 After ^b^	MR 2 After ^c^	MR 3 After ^d^
MR 1 before ^b^	1	3	0	0
MR 2 before ^c^	0	19	2	0
MR 3 before ^d^	0	6	8	2

^a^ without MR, ^b^ mild MR, ^c^ moderate MR, ^d^ severe MR. Abbreviations: HBP, His bundle pacing; MR, mitral regurgitation.

**Table 5:** Impact of medications and comorbidities on LVEF improvement, LVEDD reduction, and MR changes.

Factor	Median LVEF Improvement (%) [IQR]	*p*-Value ^a^	Change in Mean LVEDD (mm) ± SD	*p*-Value ^b^	Mean Change in MR [IQR]	*p*-Value ^c^	Effect on MR Improvement (β)	*p*-Value ^d^
Digoxin (yes vs. no)	14.6 [9.1–20.0] vs. 14.9 [9.1–20.0]	0.1495	55.6 ± 1.8 vs. 55.6 ± 1.8	0.9831	1 [0–2] vs. 1 [0–2]	0.5846	2.33	0.3145
Metoprolol (dose)	–	ρ = 0.25, *p* = 0.1091 ^e^	–	r = 0.19, *p* = 0.2437 ^e^	–	ρ = 0.02, *p* = 0.9021 ^e^	−0.01	0.3392
MRA (yes vs. no)	14.6 [9.1–20.0] vs. 14.9 [9.1–20.0]	0.7662	55.6 ± 1.8 vs. 55.6 ± 1.8	0.9308	1 [0–2] vs. 1 [0–2]	0.9308	−0.03	0.2105
IHD (yes vs. no)	10.0 [7.8–14.6] vs. 14.9 [9.1–20.0]	0.1053	55.5 ± 1.8 vs. 55.7 ± 1.8	0.8175	1 [0–2] vs. 1 [0–2]	0.1634	−0.40	0.7512
DM (yes vs. no)	14.6 [9.1–20.0] vs. 14.9 [9.1–20.0]	0.6184	56.1 ± 1.8 vs. 55.4 ± 1.8	0.3022	1 [0–2] vs. 1 [0–2]	0.7187	1.02	0.4786
CKD (yes vs. no)	14.6 [9.1–20.0] vs. 14.9 [9.1–20.0]	0.9190	55.7 ± 1.8 vs. 55.6 ± 1.8	0.9831	1 [0–2] vs. 1 [0–2]	0.6024	−4.18	0.0486
HT (yes vs. no)	14.6 [9.1–20.0] vs. 14.9 [9.1–20.0]	0.4662	55.8 ± 1.8 vs. 54.8 ± 1.8	0.4141	1 [0–2] vs. 1 [0–2]	0.6785	−16.79	0.9951

^a^ *p*-values for LVEF improvement were derived from the Mann–Whitney U test (Wilcoxon test). ^b^ *p*-values for LVEDD were derived from Student’s *t*-test. ^c^ *p*-values for final MR severity were derived from the Kruskal–Wallis test. ^d^ Logistic regression was used to model the likelihood of MR improvement (β—log-odds). ^e^ Spearman/Pearson correlation was used for metoprolol dosage and LVEF/LVEDD, respectively. Abbreviations: CKD, chronic kidney disease; DM, diabetes mellitus; LVEF, left ventricular ejection fraction; Hb, hemoglobin; HT, hypertension; IHD, ischemic heart disease; K+, potassium; LVEDD, left ventricular end-diastolic diameter; MI, myocardial infarction; MR, mitral regurgitation; MRA, mineralocorticoid receptor antagonist.

## Data Availability

The data presented in this study are available on request from the corresponding author (the data are not publicly available due to privacy or ethical restrictions).

## Abbreviations

The following abbreviations are used in this manuscript:

AF	Atrial fibrillation
AFMR	Atrial functional mitral regurgitation
AV	Atrioventricular
AVNA	Atrioventricular node ablation
BVP	Biventricular Pacing
CKD	Chronic kidney disease
CS	Coronary sinus
CRT	Cardiac resynchronization therapy
DM	Diabetes mellitus
EF	Ejection fraction
Hb	Hemoglobin
HF	Heart failure
HFH	Heart-failure-related hospitalization
HBP	His-bundle pacing
HFmrEF	Heart failure with mildly reduced
HFpEF	Heart failure with preserved ejection fraction
HFrEF	Heart failure with reduced ejection fraction
HT	Hypertension
IHD	Ischemic heart disease
K+	Potassium
LBBB	Left bundle branch block
LVEDD	Left ventricular end-diastolic diameter
LVEF	Left ventricular ejection fraction
MI	Myocardial infarction
MR	Mitral regurgitation
MRA	Mineralocorticoid receptor antagonist
PICM	Pacing-induced cardiomyopathy
RVP	Right ventricular pacing
SND	Sinus node dysfunction

## References

[1] Kotecha D., Lam C.S.P., Van Veldhuisen D.J., Van Gelder I.C., Voors A.A., Rienstra M. (2016). Heart Failure With Preserved Ejection Fraction and Atrial Fibrillation. J. Am. Coll. Cardiol..

[2] Espnes H., Wilsgaard T., Ball J., Løchen M.-L., Njølstad I., Schnabel R.B., Gerdts E., Sharashova E. (2025). Heart Failure in Atrial Fibrillation Subtypes in Women and Men in the Tromsø Study. JACC Adv..

[3] Newman J.D., O’Meara E., Böhm M., Savarese G., Kelly P.R., Vardeny O., Allen L.A., Lancellotti P., Gottlieb S.S., Samad Z. (2024). Implications of Atrial Fibrillation for Guideline-Directed Therapy in Patients with Heart Failure. J. Am. Coll. Cardiol..

[4] Kalyanasundaram A., Li N., Hansen B.J., Zhao J., Fedorov V.V. (2019). Canine and Human Sinoatrial Node: Differences and Similarities in the Structure, Function, Molecular Profiles, and Arrhythmia. J. Vet. Cardiol..

[5] Liang J.J., Silvestry F.E. (2016). Mechanistic Insights into Mitral Regurgitation Due to Atrial Fibrillation: “Atrial Functional Mitral Regurgitation”. Trends Cardiovasc. Med..

[6] Zoghbi W.A., Levine R.A., Flachskampf F., Grayburn P., Gillam L., Leipsic J., Thomas J.D., Kwong R.Y., Vandervoort P., Chandrashekhar Y. (2022). Atrial Functional Mitral Regurgitation. JACC Cardiovasc. Imaging.

[7] Calvert P., Farinha J.M., Gupta D., Kahn M., Proietti R., Lip G.Y.H. (2022). A Comparison of Medical Therapy and Ablation for Atrial Fibrillation in Patients with Heart Failure. Expert Rev. Cardiovasc. Ther..

[8] Parkash R., Wells G.A., Rouleau J., Talajic M., Essebag V., Skanes A., Wilton S.B., Verma A., Healey J.S., Sterns L. (2022). Randomized Ablation-Based Rhythm-Control Versus Rate-Control Trial in Patients with Heart Failure and Atrial Fibrillation: Results from the RAFT-AF Trial. Circulation.

[9] Chen M., Dong Z., Zhang Y., Liu J., Zhang J. (2023). A Conversion CRT Strategy Combined with AVJA May Be a Perspective Alternative for Heart Failure Patients with Persistent Atrial Fibrillation. Heart Fail. Rev..

[10] Glikson M., Nielsen J.C., Kronborg M.B., Michowitz Y., Auricchio A., Barbash I.M., Barrabés J.A., Boriani G., Braunschweig F., Brignole M. (2021). 2021 ESC Guidelines on Cardiac Pacing and Cardiac Resynchronization Therapy. Eur. Heart J..

[11] Ciesielski A., Boczar K., Siekiera M., Gajek J., Sławuta A. (2022). The Clinical Utility of Direct His-Bundle Pacing in Patients with Heart Failure and Permanent Atrial Fibrillation. Acta Cardiol..

[12] Tang J., Kong N.W., Beaser A., Aziz Z., Yeshwant S., Ozcan C., Tung R., Upadhyay G.A. (2024). Clinical Outcomes of Conduction System Pacing Compared to Biventricular Pacing in Patients with Mid-Range Ejection Fraction. J. Interv. Card. Electrophysiol..

[13] Sanders D.J., Krishnan K. (2021). Patient Selection for Biventricular Cardiac Resynchronization Therapy, His Bundle Pacing, and Left Bundle Branch Pacing. Curr. Cardiovasc. Risk Rep..

[14] Slotwiner D.J., Raitt M.H., Del-Carpio Munoz F., Mulpuru S.K., Nasser N., Peterson P.N. (2019). Impact of Physiologic Pacing versus Right Ventricular Pacing among Patients with Left Ventricular Ejection Fraction Greater than 35%: A Systematic Review for the 2018 ACC/AHA/HRS Guideline on the Evaluation and Management of Patients with Bradycardia and Cardiac Conduction Delay. Heart Rhythm..

[15] Keene D., Shun-Shin M.J., Arnold A.D., March K., Qureshi N., Ng F.S., Tanner M., Linton N., Lim P.B., Lefroy D. (2020). Within-patient Comparison of His-bundle Pacing, Right Ventricular Pacing, and Right Ventricular Pacing Avoidance Algorithms in Patients with PR Prolongation: Acute Hemodynamic Study. Cardiovasc. Electrophysiol..

[16] Ma P., Yang Y., Dai B., Zhang R., Wang N., Li D., Yin X., Gao L., Xia Y., Yang Y. (2021). Brady-arrhythmias in Patients with Atrial Fibrillation and Heart Failure of Reduced Ejection Fraction: Is His-bundle Pacing Superior to Biventricular Pacing?. Pacing Clin. Electrophysiol..

[17] Gardas R., Golba K.S., Loboda D., Biernat J., Soral T., Kulesza P., Sajdok M., Zub K. (2024). The Usefulness of His Bundle Pacing in a Heterogeneous Population of Patients with Impaired Left Ventricular Systolic Function. Cardiol. J..

[18] Yücel G., Fastner C., Hetjens S., Toepel M., Schmiel G., Yazdani B., Husain-Syed F., Liebe V., Rudic B., Akin I. (2022). Impact of Baseline Left Ventricular Ejection Fraction on Long-term Outcomes in Cardiac Contractility Modulation Therapy. Pacing Clin. Electrophysiol..

[19] Deferm S., Bertrand P.B., Verbrugge F.H., Verhaert D., Rega F., Thomas J.D., Vandervoort P.M. (2019). Atrial Functional Mitral Regurgitation. J. Am. Coll. Cardiol..

[20] Farhan S., Silbiger J.J., Halperin J.L., Zhang L., Dukkipati S.R., Vogel B., Kini A., Sharma S., Lerakis S. (2022). Pathophysiology, Echocardiographic Diagnosis, and Treatment of Atrial Functional Mitral Regurgitation. J. Am. Coll. Cardiol..

[21] Kouris N.T., Kostakou P.M., Tryfou E.S., Olympios C.D. (2023). Incidence and Causal Association of Functional Atrial Mitral Regurgitation in HFpEF. Hell. J. Cardiol..

[22] Upadhyay G.A., Henry M., Genovese D., Desai P., Lattell J., Wey H., Besser S.A., Aziz Z., Beaser A.D., Ozcan C. (2021). Impact of Physiological Pacing on Functional Mitral Regurgitation in Systolic Dysfunction: Initial Echocardiographic Remodeling Findings after His Bundle Pacing. Heart Rhythm O2.

[23] Qu Q., Sun J., Zhang Z., Kan J., Wu L., Li F., Wang R. (2021). His-Purkinje Conduction System Pacing: A Systematic Review and Network Meta-analysis in Bradycardia and Conduction Disorders. Cardiovasc. Electrophysiol..

[24] Payne J., Garlitski A.C., Weinstock J., Homoud M., Madias C., Estes N.A.M. (2018). His Bundle Pacing. J. Interv. Card. Electrophysiol..

[25] Sashida Y., Mori F., Arashi H., Hosaka F., Itai T., Ohnishi S. (2006). Improvement of Left Ventricular Function by Permanent Direct His-Bundle Pacing in a Case with Dilated Cardiomyopathy. J. Arrhythmia.

[26] Soral T., Gardas R., Gołba K.S., Kulesza P., Biernat J., Łoboda D. (2024). His Bundle Pacing Is Continually Relevant for Patients with Atrial Fibrillation and Bradycardia without Prior Atrioventricular Nodal Ablation, Data from Mid-Term Follow-Up. Pol. Heart J..

[27] Fernandes G.C., Knijnik L., Lopez J., Rivera M., Fernandes A., Lambrakos L.K., Myerburg R.J., Mitrani R.D., Goldberger J.J. (2020). Network Meta-analysis of His Bundle, Biventricular, or Right Ventricular Pacing as a Primary Strategy for Advanced Atrioventricular Conduction Disease with Normal or Mildly Reduced Ejection Fraction. Cardiovasc. Electrophysiol..

[28] Skonieczny B., Gajek A., Strózik P., Zawadzki J., Adamowicz J., Gajek J., Sławuta A. (2018). The Optimal Management of Patient with Permanent Atrial Fibrillation and Heart Failure with Reduced Ejection Fraction—The Permanent His-Bundle Pacing Is a Solution. A Case Report. J. Electrocardiol..

[29] McMurray J.J.V., Van Veldhuisen D.J. (2014). β Blockers, Atrial Fibrillation, and Heart Failure. Lancet.

[30] Kotecha D., Holmes J., Krum H., Altman D.G., Manzano L., Cleland J.G.F., Lip G.Y.H., Coats A.J.S., Andersson B., Kirchhof P. (2014). Efficacy of β Blockers in Patients with Heart Failure plus Atrial Fibrillation: An Individual-Patient Data Meta-Analysis. Lancet.

[31] Rienstra M., Damman K., Mulder B.A., Van Gelder I.C., McMurray J.J.V., Van Veldhuisen D.J. (2013). Beta-Blockers and Outcome in Heart Failure and Atrial Fibrillation. JACC Heart Fail..

[32] Mareev Y., Cleland J.G.F. (2015). Should β-Blockers Be Used in Patients With Heart Failure and Atrial Fibrillation?. Clin. Ther..

[33] Cullington D., Goode K.M., Zhang J., Cleland J.G.F., Clark A.L. (2014). Is Heart Rate Important for Patients With Heart Failure in Atrial Fibrillation?. JACC Heart Fail..

[34] Thiyagarajah A., Lau D.H., Sanders P. (2018). Atrial Fibrillation and Conduction System Disease: The Roles of Catheter Ablation and Permanent Pacing. J. Interv. Card. Electrophysiol..

[35] Amir T., Ilan M., Fishman E., Michowitz Y., Khalameizer V., Katz A., Glikson M., Medina A., Rav Acha M. (2020). “Preventive” Pacing in Patients with Tachy-brady Syndrome (TBS): Confirming a Common Practice. Int. J. Clin. Pract..

